# Passive exercise provides a simultaneous and postexercise executive function benefit

**DOI:** 10.3389/fcogn.2024.1334258

**Published:** 2024-05-20

**Authors:** Connor Dalton, Chloe Edgar, Benjamin Tari, Matthew Heath

**Affiliations:** ^1^School of Kinesiology, Faculty of Health Sciences, University of Western Ontario, London, ON, Canada; ^2^Canadian Centre for Activity and Aging, University of Western Ontario, London, ON, Canada; ^3^Graduate Program in Neuroscience, University of Western Ontario, London, ON, Canada

**Keywords:** antipointing, cognition, cortical hemodynamics, inhibitory control, oculomotor

## Abstract

**Introduction:**

Passive exercise involves limb movement via an external force and is an intervention providing an immediate postexercise executive function (EF) benefit. It is, however, unknown whether EF is improved simultaneous with passive exercise—a salient question given the advent of passive (and active) exercise workstations designed to enhance productivity and wellbeing for individuals engaged in sedentary occupations.

**Methods:**

Here, participants (*N* = 23) completed separate 20-min conditions involving active (i.e., via volitional muscle activation) and passive (i.e., via mechanically driven cycle ergometer) cycle ergometry and a non-exercise control condition. EF was assessed prior to (i.e., preintervention), simultaneous with, and immediately after (post-intervention) each condition via the antipointing task. Antipointing involves a goal-directed limb movement mirror-symmetrical to a target and is an ideal tool for the current investigation given that the task is mediated via EF inhibitory control networks that show response-dependent changes following a single bout of exercise.

**Results and discussion:**

Results showed that passive exercise produced a simultaneous and post-intervention reduction in antipointing reaction time (RT), whereas active exercise selectively produced a post-intervention—but not simultaneous—RT reduction. Thus, passive and active exercise elicited a postexercise EF benefit; however, only passive exercise produced a simultaneous benefit. That passive—but not active—exercise produced a simultaneous benefit may reflect that the intervention provides the necessary physiological or psychological changes to elicit improved EF efficiency without the associated dual-task cost(s) of volitional muscle activity.

## 1 Introduction

Executive function (EF) includes the core components of inhibitory control, working memory and cognitive flexibility and is a cognitive construct essential for activities of daily living (Miyake et al., 2000; Diamond, 2013). Extensive literature has shown that a single bout of active (i.e., via volitional muscle activation) aerobic and/or resistance exercise provides a *postexercise* EF benefit (for meta-analyses see, Lambourne and Tomporowski, 2010; Chang et al., 2012; Ludyga et al., 2016) that can persist for up to 60-min (Joyce et al., 2009; Hung et al., 2013; Shukla and Heath, 2022). A candidate mechanism for the benefit is an exercise-mediated increase in cerebral blood flow (CBF) (for review see, Smith and Ainslie, 2017) that improves EF network efficiency via thermo-mechanical changes to the brain's glial and neural networks (i.e., the hemo-neural hypothesis) (Moore and Cao, 2008). More specifically, an exercise-mediated increase in CBF is proposed to improve neurovascular coupling and cerebral perfusion (for review see, Girouard and Iadecola, 2006) that enhance the efficiency of local neural circuits via vessel diameter changes specific to pial and penetrating arterioles (i.e., mechanical change) and improved temperature regulation (i.e., cooling) (for extensive review see, Nippert et al., 2018).

Passive exercise entails movement of the limbs via an external mechanical force and is a technique known to increase CBF independent of volitional muscle activation or increased cardiorespiratory demands. Unlike active exercise, wherein the primary mechanism of a rise in CBF is an increase in arterial carbon dioxide (PaCO_2_) (for review see, Ogoh and Ainslie, 2009), passive exercise increases CBF via activation of mechanoreceptive type *III* muscle afferents to somatosensory and primary motor cortices that increase cardiac output and stroke volume (Nóbrega and Araujo, 1993). Notably, Shirzad et al. (2022) showed that a passive exercise increase in CBF was linked to a postexercise EF benefit. In particular, Shirzad et al. had participants complete 20-min sessions of light intensity active (i.e., 70 rpm at 30 W) and passive (i.e., mechanically driven flywheel set a 70 rpm) cycle ergometry as well as a non-exercise control condition. During all interventions, transcranial Doppler ultrasound (TCD) measured middle cerebral artery velocity (MCAv) to estimate CBF, and EF was assessed prior to and immediately following each condition via an inhibitory control EF task (see details below). As expected, the control condition did not impact MCAv nor EF, whereas passive and active exercise produced a baseline to steady state increase in MCAv—albeit with a larger change in the latter condition—and both conditions produced a postexercise inhibitory control EF benefit. Further, Tari et al. (2023) showed that the passive exercise benefit was limited to the first 20-min postexercise, whereas active exercise provided a more persistent (i.e., ~60 min) benefit.

A question not addressed by Shirzad et al. (2022) is whether an EF benefit is accrued when assessed simultaneous with passive exercise. This is a salient question given the advent of active and passive workstations that are thought to improve productivity and cognitive engagement for persons in sedentary occupations (Zhang et al., 2018). This question also provides a basis to evaluate whether a simultaneous exercise-based increase in CBF is linked to any change in EF efficiency and effectiveness. Moreover, the need to evaluate this issue is highlighted by equivocal findings from the limited work examining inhibitory control simultaneous with active exercise (for meta-analysis see, Zheng et al., 2021). The literature has reported that 11 min or more (typical duration of 20 min) of light to moderate intensity active exercise can improve (Joyce et al., 2009; Ogoh et al., 2014), impair (Del Giorno et al., 2010; Olson et al., 2016) or not impact (Davranche et al., 2009; McMorris et al., 2009) the simultaneous assessment of inhibitory control. Indeed, Joyce et al. (2009) reported that a 30 min moderate intensity cycling protocol improved Stop Signal reaction time (RT), whereas Olson et al. (2016) observed that a 31 min moderate intensity cycling protocol impaired Ericksen flanker task response accuracy. One explanation for the mixed findings is that light to moderate intensity active exercise does not provide the physiological steady-state change(s) (e.g., increase in CBF, catecholamines, psychological arousal) necessary to elicit a simultaneous EF benefit (Chang et al., 2012), and/or that such intensities require at least 20-min of duration before a benefit can be accrued. As such, it could be argued that assessing simultaneous EF early in the exercise intervention (i.e., within minutes after exercise onset) does provide a sufficient exercise duration to observe a benefit. This view is, however, challenged by findings showing that active exercise elicits an EF benefit across the continuum of light to very heavy intensities (e.g., Heath et al., 2018; Tari et al., 2021b; for meta-analysis see, Ludyga et al., 2016) and following as little as 10-min of exercise (Johnson et al., 2016; Samani and Heath, 2018). A second explanation is that performing an EF task simultaneous with exercise renders competition for limited attentional resources (i.e., dual-task effect) (Pashler, 1994) resulting in a performance decrement in one or both tasks (for review see, McDowd and Shaw, 2000). In support of this view, Callicott et al.'s (1999) seminal functional magnetic resonance imaging study reported that regional-specific nodes associated with EF (i.e., dorsolateral prefrontal cortex) demonstrate capacity limitations during dual-task operations, and near-infrared spectroscopy has shown that simultaneous active exercise and an EF task produce a larger cortical hemodynamic response in the prefrontal cortex than when the tasks are performed independently; such activity patterns may impede EF (Park et al., 2021). What is more, active exercise requires participants to adhere to a prescribed intensity and/or pedal frequency (for review see Zheng et al., 2021) promoting an attentional prioritization that may hinder the expression of a simultaneous EF benefit (Brisswalter et al., 1995).

Passive cycle ergometry does not require volitional muscle activation nor does it require attentional demands to a specific intensity or pedaling frequency. As a result, passive exercise provides a basis to evaluate EF independent of dual-task attentional constraints. Accordingly, we examined whether passive and active cycle ergometry paired simultaneously with an EF task influences the expression of an inhibitory control EF benefit (or decrement). Healthy young adults completed 20-min sessions of passive (70 rpm) and active (70 rpm at 25 W) cycle ergometry as well as a non-exercise control condition. The work rate for the active exercise intervention was selected for three reasons. First, previous work by our group (Tari et al., 2021b) employing the same light intensity protocol reported a reliable baseline to steady state increase in CBF—as estimated via MCAv—and produced a postexercise EF benefit equivalent in magnitude to moderate and heavy intensity work rates (see also Heath et al., 2018; Petrella et al., 2019). Second, pilot testing demonstrated that light intensity exercise at 25 W is preferrable to no-load cycling because maintaining a constant pedal cadence in the latter task is attentionally demanding and resulted in frequent postural adjustments. Third, the light intensity work rate used here provides a reasonable proxy for CBF changes associated with passive exercise. EF was assessed prior to (i.e., pre-intervention), during (i.e., simultaneous) and following (i.e., post-intervention) each condition via pro- (i.e., point to veridical location of exogenously presented target) and antipointing (i.e., point to mirror-symmetrical to a target) trials. Antipointing produces longer reaction times (RTs) and decreased endpoint accuracy compared to propointing and these behavioral “costs” have been linked to the EF demands of supressing a propointing response (i.e., inhibitory control) and the 180° spatial transposition of a target's coordinate's (i.e., vector inversion) (Carey et al., 1996; Heath et al., 2009; Maraj and Heath, 2010; for review of antisaccades see, Munoz and Everling, 2004). Thus, RT performance during the antipointing task has been taken to evince a manual-motor index of the inhibitory control component of EF. Moreover, the EF networks supporting antipointing (Connolly et al., 2000; Heath et al., 2011) overlap with prefrontal regions showing task-dependent changes following a single bout of exercise (Herold et al., 2020). As such, pro- and antipointing provide a basis to determine whether passive and active exercise elicit a general information processing benefit (i.e., decreased pro- and antipointing RTs) or a selective EF benefit (i.e., decreased antipointing—but not propointing—RTs). In addition to behavioral measures, transcranial Doppler ultrasound (TCD) of middle cerebral artery velocity (MCAv) was measured to estimate whether passive and active exercise CBF changes are linked to simultaneous or post-intervention EF benefits. In terms of research predictions, if passive—but not active—exercise provides a reduction in antipointing RTs during the simultaneous assessment then results would support the assertion that volitional muscle activation and/or task-based attention demands (i.e., active exercise) contributes to a dual-task interference hindering the expression of an exercise-mediated EF inhibitory control benefit. In contrast, if passive and active exercise produce a simultaneous reduction in antipointing RTs then results would evince that both conditions provide the requisite physiological and/or psychological adaptation/state supporting the improved inhibitory control component of EF.

## 2 Materials and methods

### 2.1 Participants

Twenty-three participants (11 female) with an average age of 22.4 years (SD = 2.5; range: 19–26 years) were recruited from the University of Western Ontario community. Sample-size was determined *a priori* via an effect size derived from a previous study (Shirzad et al., 2022) examining passive and active exercise changes in an inhibitory control measure of EF (α = 0.05, power = 0.90, *d*_*z*_ = 0.75). Participants self-reported being right-handed (i.e., “What hand do you write with?”) with normal or corrected-to-normal vision, no history of smoking, cardiorespiratory disease, neurological/neuropsychiatric disease (including concussion) and reported not taking medication influencing cardiovascular, hemodynamic, or metabolic responses to exercise. Participants were requested to refrain from alcohol, recreational drugs and caffeine 12 h prior to a study session and get 8 h of sleep the night before a study session—all participants reported adhering to these requests. Data collection occurred between 9:00 and 11:00 a.m. in a hydrated state (i.e., ~555 ml of water consumed 60-min in advance of data collection). Participants read a letter of information and signed a consent form approved by the Health Sciences Research Ethics Boards, University of Western Ontario (HSREB #120489). The study was conducted in accord with the most recent Declaration of Helsinki with the exception that participants were not entered into a database.

All participants obtained a full score on the 2020 Physical Activity Readiness Questionnaire (PAR-Q+) (Warburton et al., 2011) indicating their ability to complete an exercise intervention and completed the Godin Leisure-Time Exercise Questionnaire (GLTEQ) (Godin, 2011). All participants indicated “No” for the first seven questions of the PAR-Q and thus indicated that participants were ready to participate in physical activity and did not have a chronic medical condition nor any condition requiring chronic medication. The group mean GLETQ score was 62 (SD = 18, range = 31–95) indicating that all participants were recreationally active. We did not collect a direct measure of participants' cardiorespiratory fitness given evidence from a recent meta-analysis reporting that a single bout exercise benefit to EF is observed across the continuum of healthy young adults deemed low- to high-fit (Ludyga et al., 2016).

### 2.2 Apparatus and procedures

**Figure 1:**
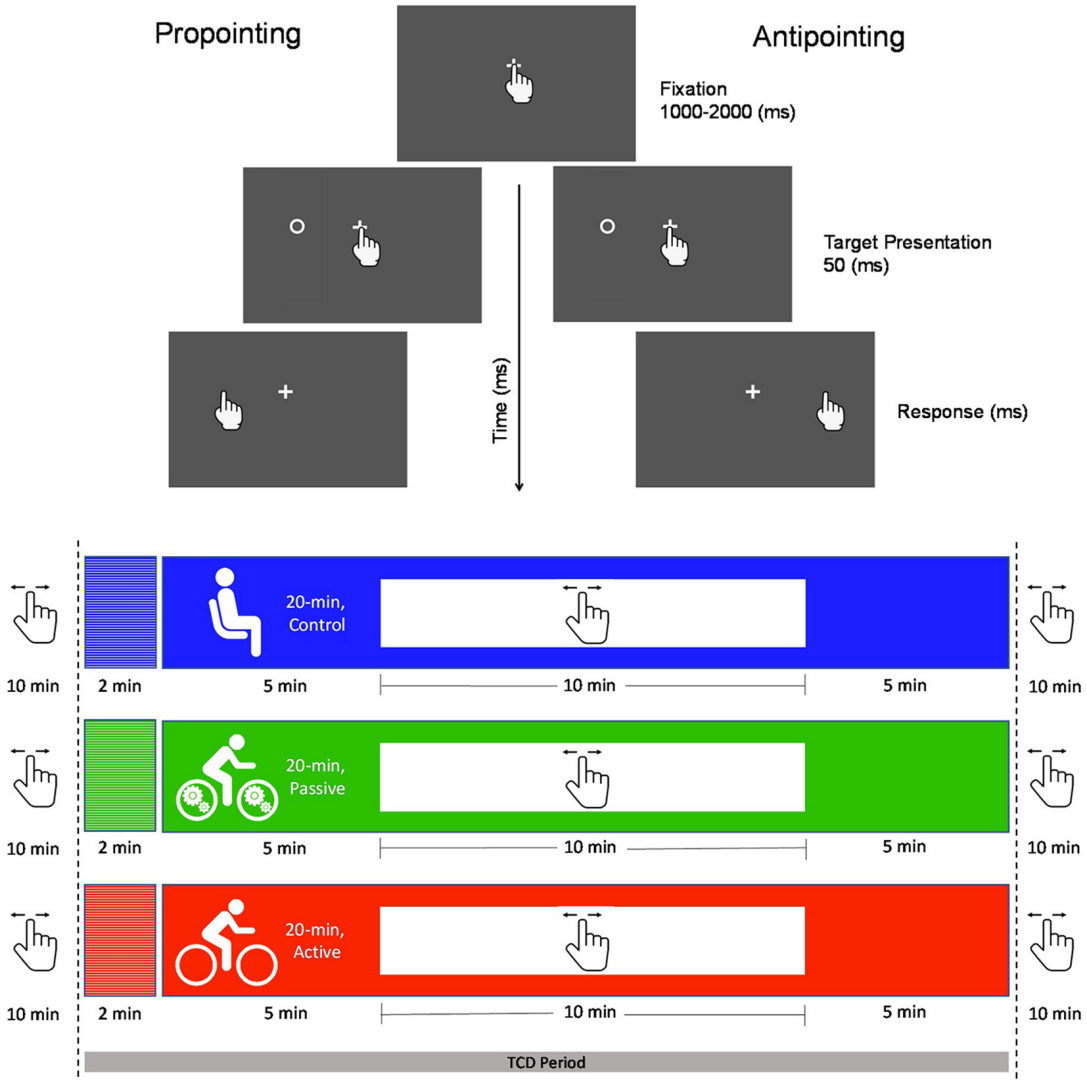
The **top pane**l provides a schematic of the pro- (i.e., point to veridical target location) and antipointing (i.e., point mirror-symmetrical to target location) trials used here. For each trial, a fixation cross was presented and cued participants to place their pointing finger (i.e., right index) on its location which initiated a randomized foreperiod after which a target was briefly (50 ms) presented left or right of fixation. The fixation cross remained visible throughout a trial (i.e., overlap paradigm). Target onset cued participants to pro- or antipoint “as quickly and accurately as possible”. The **bottom panel** shows the timeline of pro- and antipointing assessments in the passive exercise, active exercise and control conditions and indicates when transcranial Doppler ultrasound (TCD) was used to measure middle cerebral artery velocity (MCAv).

Three conditions (i.e., control, passive exercise and active exercise) were completed on separate days separated by at least 24 h with order determined via MATLAB's (R2018a; The MathWorks, Natick, MA, USA) randomization function. The randomization resulted in 6, 8, and 9 participants completing the control, passive and active conditions, respectively, as their first experimental sessions and 9, 7 and 7 participants completing the control, passive and active exercise conditions, respectively, as their last experimental session. For all conditions, participants sat upright in front of a table (height = 82 cm, depth = 76 cm, width = 106 cm) on an active-passive cycle ergometer (E-PAT AP; Healthcare International, Langley, WA, USA) equipped with a mechanically driven flywheel and their feet secured to the ergometer pedals via Velcro straps. Participants were positioned such that their legs achieved ~85% of extension at the end of a pedal stroke and so that their arms could be comfortably placed on the tabletop. All conditions were preceded by a 10-min baseline wherein participants remained stationary on the ergometer (for schematic see, Figure 1). After baseline in the control condition, participants remained stationary on the ergometer for an additional 20-min. In contrast, after baseline in the passive and active exercise conditions participants step-transitioned to 20-min of passive or active exercise. For passive exercise, the 20-min intervention entailed participants having their legs cycled at 70 rpm via a mechanically driven flywheel. During this time participants were instructed to not actively engage their leg muscles and previous electromyography work has shown that this protocol does not produce volitional muscle activation in the lower limbs (Shirzad et al., 2022). For active exercise, the 20-min intervention entailed a 70 rpm cadence at 25 W and thus represented “light” intensity in the exercise work-rate continuum (Warburton et al., 2006). A metronome (MA-2-BKRD; Korg, Tokyo, Japan) was used in all conditions to assist in cadence maintenance. For all conditions, EF was assessed via the pro- and antipointing task (see details below) at: (1) 10-min prior to the onset of each intervention (i.e., pre-intervention), (2) 5-min following the onset of each intervention (i.e., simultaneous), and (3) immediately following each intervention (i.e., post-intervention) (Figure 1).

During baseline and intervention, heart rate (HR) was monitored (Polar Electro T34, Kempele, Findland) using PowerLab (ML132/ML880, ADInstruments, Colorado Springs, CO, USA) and calculated (using a 5 s rolling average) based on successive heart beats (i.e., RR interval). TCD data during baseline and intervention were collected via a probe (Neurovision 500M, Neurovision TOC2M; Multigon Industries, Elmsford, CA) coated in aqueous ultrasound gel (Aquasonic Clear, Parker Laboratories Inc., Fairfield, NJ) and secured to participants' right anterior temporal window via an adjustable headband. TCD provided a measure of middle cerebral artery velocity (MCAv) and is a technique Bishop et al. (1986) have shown to provide a valid proxy to a direct measure of CBF (i.e., Xenon tracing of the MCA). Moreover, MCAv has been used extensively in the exercise neuroscience/physiology literature as a valid estimate for exercise-mediated changes in CBF (Jørgensen et al., 1985; for meta-analysis see, Smith et al., 2021).

### 2.3 Executive function assessment

Pre-intervention, simultaneous-intervention and post-intervention assessments of the inhibitory control component of EF were completed via pro- (i.e., point to veridical target location) and antipointing (i.e., point to mirror-symmetrical target location) trials (Figure 1). Hence, across the control, passive and active exercise conditions nine EF assessments were completed. Pro- and antipointing trials were completed on a computer tablet (12.9′inch iPad^®^ Pro, OSX v. 15.6.1) running a custom-built app (XCode developed via Swift; v. 5.3 Apple Inc, Cupertino CA) operating at a native screen and touch resolution of 60 Hz (for details see, Tari and Heath, 2021). Participants were instructed on the nature of pro- and antipointing trials by the experimenters. During data collection the computer tablet was secured to the tabletop 40 cm in front of participants' midline in landscape mode and oriented with a positive anteroposterior angle of 10°. Visual stimuli were presented on a gray (RGB code: 125, 125, 125) background (17 cd/m^2^) and included a centrally located white (RGB code: 255, 255, 255) home location (i.e., 1 by 1 cm cross, 37 cd/m^2^) and targets (i.e., open white circle; 1 cm in diameter, 37 cd/m^2^) presented 6 cm (i.e., proximal target) and 9 cm (i.e., distal target) left and right of the home location and in the same horizontal plane. The beginning of a trial was denoted by onset of the home location which instructed participants to place their right index finger (i.e., the pointing finger) on its location. Contact with the home location initiated a uniformly distributed randomized foreperiod (1,000 and 2,000 ms) after which a target appeared (temporal overlap with the home location cue) for 50 ms in one of four locations (i.e., left 6 or 9 cm; right 6 or 9 cm). Target onset cued participants to pro- or antipoint “quickly and accurately” and participants were instructed to “lift and point”—as opposed to slide—to their final pointing location. Pro- and antipointing trials were completed in separate and randomly ordered blocks wherein 20 trials were pseudorandomly presented at each target location (i.e., left and right field) and eccentricity (i.e., proximal and distal) for a total of 160 total trials. Prior to a block of trials an instruction screen indicated the nature (i.e., pro- vs. antipointing) of the upcoming series of trials. Approximately 10-min were required to complete each pro- and antipointing session. The EF assessment was initiated at the 5-min mark of the simultaneous and post-intervention sessions.

### 2.4 Data reduction, dependent variables, and statistical analyses

TCD data corrupted by signal aliasing and/or signal loss (e.g., a sudden head shift) were omitted (Terslev et al., 2017) and systolic MCAv were retained for analysis (Clyde et al., 1996). Peak systolic values were analyzed given Rosengarten and Kaps (2002) demonstration that they provide a valid measure for TCD-based changes to MCAv and index task-based demands in countermanding tasks (Duschek et al., 2018; Tari et al., 2021a). For each condition, mean values were determined from the last minute of baseline and the last minute of intervention (i.e., steady-state) (Figure 1). HR and MCAv data were examined via 3 (**condition**: control, passive exercise, active exercise) by 2 (**time**: baseline, steady-state) fully repeated measures ANOVA.

Pro- and antipointing dependent variables included RT (i.e., time from target onset to release of pressure from home location), movement time (MT: i.e., time from release of pressure from home location to a touch of the tablet screen) and horizontal endpoint gain variability (i.e., within-participant standard deviation of movement amplitude/veridical target amplitude). As per previous work (Maraj and Heath, 2010; Tari and Heath, 2021) RTs <150 ms (i.e., anticipatory response) or 2.5 times a participant- and task-specific mean were excluded as were trials involving a directional error (i.e., a propointing response instead of an instructed antipointing response and *vice versa*). In turn, trials involving a MT <100 ms or 2.5 times a participant- and task-specific mean were excluded. Less than 5% of trials for any participant were excluded and this is accounted for by the use of a randomly distributed foreperiod and the use of an overlap paradigm involving the blocked presentation of pro- and antipointing trials. RT, MT and gain variability were examined via 3 (**condition**: control, passive exercise, active exercise) by 3 (**time**: pre-intervention, simultaneous, post-intervention) by 2 (**task**: pro-, antipointing) fully repeated measures ANOVAs. Interactions were decomposed via reduced model ANOVAs and simple effects (i.e., single sample *t*-tests). Where appropriate: Huynh-Feldt corrections for violations of sphericity are reported (i.e., degrees of freedom adjusted to one decimal place), and two one-sided test (TOST) statistics (*d*_*z*_ = 0.50) were employed to determine if values were within an equivalence boundary (for tutorial see Lakens, 2017). Further, and where appropriate, Bayesian single-sample *t*-tests were used to quantify evidence in favor of the null hypothesis (for tutorial see, Faulkenberry et al., 2020).

## 3 Results

### 3.1 Heart rate and middle cerebral artery velocity

HR produced main effects of **condition**, *F*_(2, 44)_ = 7.07, *p* = 0.002, $\eta_{\text{p}}^{2}$ = 0.24, **time**, *F*_(1, 22)_ = 36.64, *p* < 0.001, $\eta_{\text{p}}^{2}$ = 0.62, and their interaction, *F*_(2, 44)_ = 23.81, *p* < 0.001, $\eta_{\text{p}}^{2}$ = 0.52. The interaction was decomposed by computing participant-specific HR difference scores (i.e., steady-state minus baseline) and contrasting to a value of zero via single-sample *t*-tests. Control (0 bpm, SD = 4) and passive exercise (−1 bpm, SD = 5) difference scores did not reliably differ from baseline to steady-state [ts_(22)_ = 0.55 and −1.06 for control and passive exercise, ps = 0.55 and 0.30, *d*_*z*_ = 0.11 and −0.22], whereas active exercise produced a baseline to steady-state HR increase (14 bpm, SD = 10) [*t*_(22)_ = 6.25, *p* < 0.001, *d*_*z*_ = 1.30].

For the active exercise condition, the group average absolute steady state HR was 97 bpm (SD = 11), whereas control and passive exercise produced values of 79 (SD = 9) and 82 (SD = 9) bpm, respectively. Light intensity exercise is defined as a work rate between 45 and 54% of heart rate maximum (HR_max_: i.e., 220 minus age in years) (for description of exercise work rates see, Warburton et al., 2006) and for active exercise the average HR_max_ was 48% (SD = 6) and a single sample TOST statistic showed that group HR_max_ values were within an equivalence boundary for a light intensity work rate [*t*_(22)_ = 2.13, *p* = 0.002]. In other words, participants exercised within the prescribed light intensity work rate.

**Figure 2:**
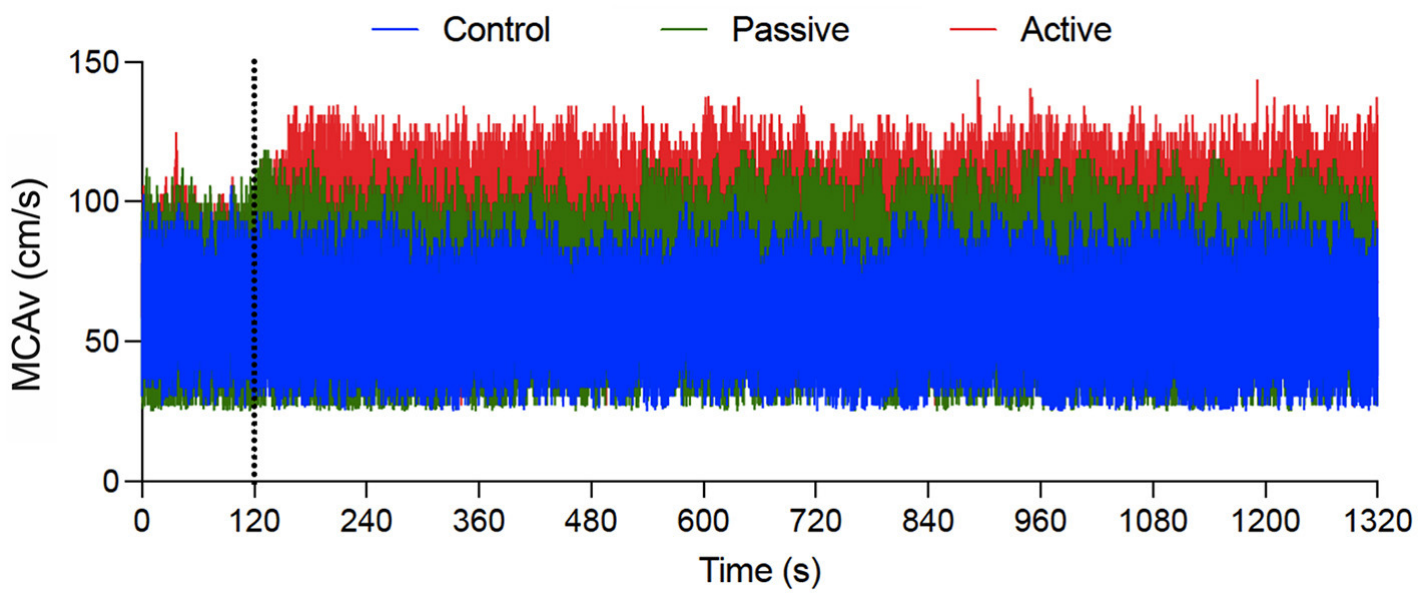
An exemplar participant's middle cerebral artery velocity (MCAv: in cm/s) for control (blue), passive exercise (green), and active exercise (red) conditions. Data are presented continuously across a 22-min window. The vertical gray dotted line indicates the transition from baseline to the exercise/control intervention.

**Figure 3:**
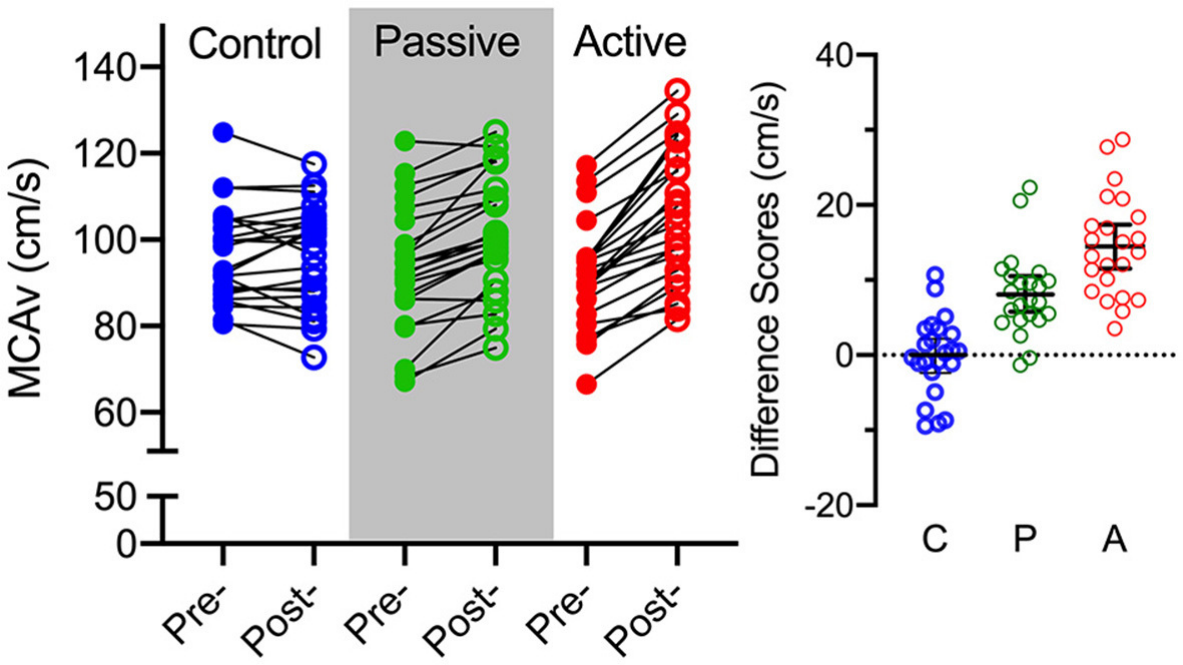
The **left panel** shows participant-specific peak systolic middle cerebral artery velocity (MCAv: in cm/s) for control (blue), passive exercise (green), and active exercise (red) conditions. The smaller **right offset panel** represents participant-specific peak systolic MCAv difference scores (steady-state minus baseline) for control (C), passive exercise (P), and active exercise (C) conditions. The **offset panel** also presents the group mean MCAv difference score (i.e., black horizontal line) and associated 95% between-participant confidence interval (i.e., blacked capped vertical line).

Figure 2 shows MCAv data for an exemplar participant as a function of the last 2 min of baseline and throughout the intervention for each condition. The figure qualitatively demonstrates that passive and active exercise—but not the control condition—produced a baseline to steady-state increase in MCAv; albeit the magnitude was larger during active exercise. In terms of quantitative analyses, MCAv produced a main effect of **time**, *F*_(1, 22)_ = 88.68, *p* < 0.001, $\eta_{\text{p}}^{2}$ = 0.80, and a **condition by time** interaction, *F*_(2, 44)_ = 41.36, *p* < 0.001, $\eta_{\text{p}}^{2}$ = 0.65. The interaction was decomposed via the same difference score approach used for HR. Figure 3 shows participant-specific peak systolic MCAv and associated MCAv difference scores separately for each condition. The difference scores demonstrate that control condition baseline and steady-state MCAv (−0.1 cm/s, SD = 5.0) did not reliably differ [*t*_(22)_ = −0.11, *p* = 0.91, *d*_*z*_ = −0.02], whereas passive (8.2 cm/s, SD = 5.4) and active (14.4 cm/s, SD = 6.1) exercise showed a baseline to steady-state increase in MCAv [ts_(22)_ = 7.13 and 10.28, for passive and active exercise conditions, respectively, ps <0.001, *d*_*z*_ = 1.48 and 2.14]. Further, a paired-samples *t*-test contrasting passive and active exercise showed that the difference score was larger in the latter condition [*t*_(22)_ = 3.67, *p* = 0.001, *d*_*z*_ = 0.76]; that is, active exercise produced a larger *magnitude* change in MCAv.

### 3.2 Executive function assessment: reaction time, movement time and gain

**Figure 4:**
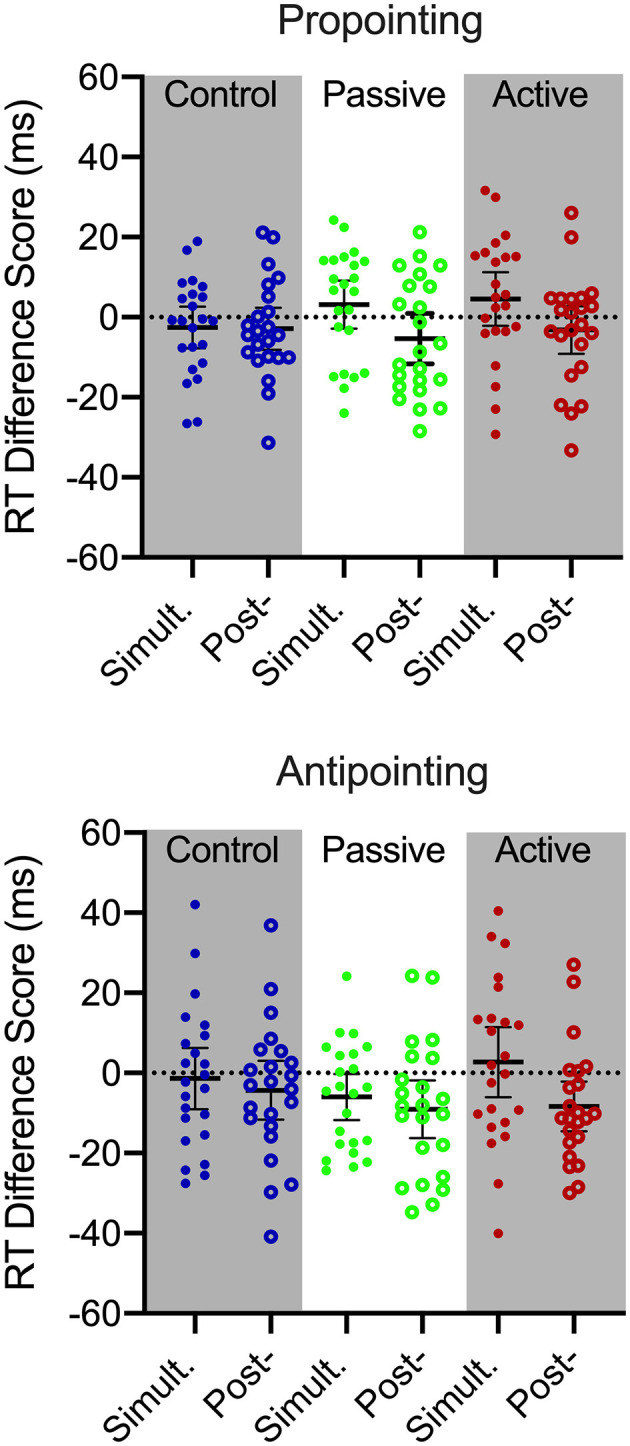
Participant-specific pro- **(top)** and antipointing **(bottom)** reaction time (RT) difference scores (Simult. = simultaneous-intervention minus pre-intervention; Post- = post-intervention minus pre-intervention) for control (blue), passive exercise (green) and active exercise conditions. Error bars represents 95% between-participant confidence intervals. The absence of overlap between error bars and zero (i.e., horizontal dotted line) indicated a reliable difference inclusive to a test of the null hypothesis.

RT yielded main effects for **time**, *F*_(2, 44)_ = 8.87, *p* < 0.001, $\eta_{\text{p}}^{2}$ = 0.29, **task**, *F*_(1, 22)_ = 57.50, *p* < 0.001, $\eta_{\text{p}}^{2}$ = 0.72, and interactions involving **time by task**, *F*_(2, 44)_ = 3.50, *p* = 0.040, $\eta_{\text{p}}^{2}$ = 0.13, and condition by time by task, *F*_(4,88)_ = 2.62, *p* = 0.040, $\eta_{\text{p}}^{2}=0.11$. To decompose the highest order interaction we computed 3 (**time**: pre-intervention, simultaneous, post-intervention) by 2 (**task**: pro-, antipointing) repeated measures ANOVAs separately for control, passive and active exercise conditions. For all conditions, the reduced model ANOVAs yielded main effects for **task**, Fs_(1, 22)_ = 61.28, 46.55, and 59.34 for control, passive and active exercise, respectively, ps <0.001, $\eta_{\text{p}}^{2}$ > 0.65, indicating that RTs for propointing (198 ms, SD = 23) were shorter than antipointing (243 ms, SD = 44). For the control condition, there was neither a main effect of **time** nor a **time by task interaction**, Fs_(2, 44)_ = 1.82 and 0.54, ps = 0.18 and 0.58, $\eta_{\text{p}}^{2}$ = 0.07 and 0.02. In turn, passive and active exercise elicited main effects for **time**, Fs_(2, 44)_ = 4.49 and 4.90 for passive and active exercise, respectively, ps = 0.017 and 0.012, $\eta_{\text{p}}^{2}$ = 0.17 and 0.18, and **time by task** interactions, Fs_(2, 44)_ = 3.28 and 3.52, ps = 0.047 and 0.038, $\eta_{\text{p}}^{2}$ = 0.13 and 0.14. To decompose the interactions, we computed RT difference scores (i.e., simultaneous minus pre-intervention; post-intervention minus pre-intervention) and contrasted to zero via single-samples *t*-tests. Figure 4 shows that propointing RT difference scores for passive and active exercise conditions at simultaneous and post-intervention assessments did not reliably differ from pre-intervention (ts_(22)_ <|1.21|, ps > 0.23, *d*_*z*_ < |0.25|). For antipointing, passive exercise difference scores indicated that simultaneous assessment values were shorter than their pre-intervention counterparts [*t*_(22)_ = −2.16, *p* = 0.042, *d*_*z*_ = −0.045], whereas active exercise simultaneous and pre-intervention difference scores did not reliably differ [*t*_(22)_ = −0.64, ps = 0.52, *d*_*z*_ = −0.16] and a TOST statistic (*d*_*z*_ = 0.55) indicated that values were within an equivalence boundary [ts_(22)_ = 1.75, *p* = 0.036]. In turn, post-intervention RT difference scores for passive and active exercise conditions were shorter than their pre-intervention counterparts [ts_(22)_ = −2.62 and −2.77, for passive and active exercise, respectively, ps = 0.016 and 0.011, *d*_*z*_ = −0.51 and −0.58]. Moreover, given the objective of this study and the mixed findings reported in the literature, we employed a Bayesian single sample *t*-statistic (Cauchy prior: *r* = 0.707) to quantify evidence for the null hypothesis for the antipointing RT difference scores contrasting active exercise simultaneous and pre-intervention values. Results produced a BF_01_ value of 3.80 and thus provides moderate support in favor of the null hypothesis (for Bayesian nomenclature see, Jeffreys, 1961).

MT and gain variability produced main effects of **task**, Fs_(1, 22)_ = 21.54 and 61.46 for MT and gain variability, respectively, ps <0.001, $\eta_{\text{p}}^{2}$ = 0.49 and 0.74, indicating that propointing MTs were shorter (177 ms, SD = 41) and endpoint less variable (0.12, SD = 0.02) than antipointing (MT: 191 ms, SD = 51, gain variability: 0.15, SD = 0.03)—a finding that was independent of condition and time. As well, MT produced a main effect of **time**, F_(2, 44)_ = 4.95, *p* = 0.18, $\eta_{\text{p}}^{2}$ = 0.16, and a **time by task** interaction, F_(2, 44)_, *p* = 0.023. $\eta_{\text{p}}^{2}$ = 0.14. To decompose the **time by task** interaction we computed participant-specific MT difference scores (simultaneous minus pre-intervention, post-intervention minus pre-intervention) separately for pro- and antipointing and contrasted results to a value of zero. Results showed that propointing MT difference scores at simultaneous (4 ms, SD = 17) and post-intervention (2 ms, SD = 15) did not differ from their pre-intervention counterparts [ts_(22)_ = 1.32 and 0.61, ps = 0.20 and 0.54, *d*_*z*_ = 0.27 and 0.13]. In contrast, antipointing MT difference scores at simultaneous (−11 ms, SD-19) and post-intervention (−10 ms, SD = 18) were less than their pre-intervention counterparts [ts_(22)_ = 2.86 and 2.64, *p* < 0.01 and 0.015, *d*_*z*_ = 0.59 and 0.55]. Notably, MT and gain variability did not elicit any other significant main effects or interactions, ps > 0.118, $\eta_{\text{p}}^{2}$ <0.094.

### 3.3 Relationship between MCAv and antipointing difference scores

We computed Pearson correlation coefficients involving passive and active exercise condition MCAv difference scores and their associated simultaneous and post-intervention antipointing RT difference scores. Results for passive (rs <0.12, ps > 0.56) and active (rs <0.12, ps > 0.26) exercise conditions difference scores for each variable were not reliably related at any timepoint.

## 4 Discussion

The primary goal of this study was to determine whether passive exercise provides a simultaneous benefit to an inhibitory control measure of EF. As a secondary goal, we measured MCAv to determine whether putative exercise-mediated EF benefits are linked to changes in CBF.

### 4.1 Heart rate and middle cerebral artery velocity in passive and active exercise

Volitional muscle activation during active exercise increases CO_2_ and NO and leads to vascular deformation, increased HR, stroke volume, cardiac output and an associated increase in CBF (for review see, Smith and Ainslie, 2017). The current study observed that active exercise increased HR consistent with a very-light intensity work rate (Norton et al., 2009) and was associated with a 14 cm/s (CI_95%_ = 4) baseline to steady-state increase in MCAv. In turn, passive exercise did not increase HR and our group's previous work has shown that passive exercise does not alter ventilation or gas exchange variables (i.e., $\dot{V\dot{\;}}$O_2_ and $\dot{V\dot{\;}}$CO_2_) (Shirzad et al., 2022). Notably, however, passive exercise produced an 8 cm/s (CI_95%_ = 4) baseline to steady-state increase in MCAv; albeit the magnitude was less than active exercise. That passive exercise increased MCAv is accounted for by the activation of group *III* mechanoreceptors from the passively moving limbs that stimulate primary somatosensory and motor cortices to increase cardiac output and stroke volume (Nóbrega and Araujo, 1993). Last, and as expected, the control condition did not produce baseline to steady-state changes in HR or MCAv.

### 4.2 Antipointing planning times evince top-down executive function

Antipointing produced longer RTs, MTs and increased endpoint variability compared to propointing—a result consistent across each condition and assessment window. The longer antipointing RTs have been attributed to the time-consuming EF demands of suppressing a prepotent propointing response (i.e., inhibitory control) (Carey et al., 1996; Heath et al., 2009). In turn, the longer MTs and more variable endpoints of antipointing reflect their mediation via visual information (i.e., relative) functionally distinct from the metrically precise visual information supporting propointing (Connolly et al., 2000; Heath et al., 2011). Thus, the distinct pro- and antipointing metrics observed here provide a basis to determine whether passive and active exercise selectively benefit a task requiring top-down EF (i.e., antipointing) or more generally improves information processing (i.e., pro- and antipointing).

### 4.3 Passive and active exercise support a postexercise executive function benefit

Although the primary goal of this work was to determine whether passive exercise provides a simultaneous EF benefit, we thought it important to first discuss our postexercise findings to demonstrate their consistency with the extant literature. Indeed, at the postexercise assessment, passive and active exercise produced antipointing—but not propointing—RTs that were shorter than their pre-exercise counterparts (i.e., 5% reduction), whereas control condition pro- and antipointing RTs did not show a difference between the same assessment windows. These results are salient for at least three reasons. First, that antipointing—but not propointing–showed a postexercise RT reduction indicates that passive and active exercise did not provide a general improvement in physiological/psychological arousal and/or information processing; after all, if that were the case then pro- *and* antipointing would have exhibited a postexercise RT reduction. Second, that the control condition did not show a change in antipointing RT indicates that the passive and active exercise condition reductions in antipointing RT cannot be tied to a practice-related performance benefit. Third, antipointing—but not propointing—showed a post-exercise (and simultaneous assessment) reduction in MT across all conditions and is a finding taken to reflect a learning-based improvement in the control of a spatially remapped movement trajectory (see Maraj and Heath, 2010). Notably, however, gain variability did not change over the same assessment window for pro- or antipointing and is a result demonstrating that the post-exercise RT benefit for the latter task cannot be accounted for by an explicit or implicit strategy designed to decrease movement planning and response execution times at the cost of increased endpoint error (i.e., speed-accuracy trade-off) (Fitts, 1954). Instead, the RT finding for the active condition is consistent with a wealth of evidence reporting that a single bout of active exercise provides an immediate postexercise EF benefit (for meta-analyses see, Lambourne and Tomporowski, 2010; Chang et al., 2012), and the passive exercise findings accord recent work by our group reporting a postexercise EF benefit (Shirzad et al., 2022; Tari et al., 2023).

### 4.4 Passive—but not active—exercise supports a simultaneous executive function benefit

Passive exercise antipointing RTs at the simultaneous assessment were shorter than their pre-intervention counterparts, whereas for the active exercise condition, null hypothesis, equivalence and Bayesian tests showed that simultaneous and pre-intervention RTs were comparable. Indeed, our statistical evidence (TOST and Bayesian *t*-tests) supporting the null hypothesis for active exercise antipointing RTs accords some literature reporting that steady state moderate intensity exercise does not provide a simultaneous inhibitory control EF benefit (for systematic review see, Zheng et al., 2021). One interpretation for the absence of a positive EF benefit during active exercise is that the exercise intensity, duration, or a combination thereof, does not provide the requisite physiological steady-state to elicit an EF benefit (e.g., Lambourne and Tomporowski, 2010; Chang et al., 2012). In contrast to this view, accumulating evidence demonstrates that active exercise durations as brief as 10-min (e.g., Johnson et al., 2016; Samani and Heath, 2018; Damrongthai et al., 2021) and spanning a continuum of metabolically sustainable intensities (Heath et al., 2018; Petrella et al., 2019; Tari et al., 2021b) improves EF. A second interpretation is that a inhibitory control measure of EF evaluated concurrent with a volitional motor task leads to task-based attentional (Ruffieux et al., 2015) and/or neural competition (Callicott et al., 1999) precluding or hindering a performance benefit for one or both tasks (for review see, McDowd and Shaw, 2000). We believe that our results support the latter view given that passive exercise produced a simultaneous inhibitory control EF benefit. Hence, we believe the present results add importantly to the literature insomuch as they demonstrate that: (1) passive exercise provides a simultaneous inhibitory control EF benefit, and (2) the absence of a reliable EF benefit when performed simultaneous with active exercise may relate to dual-task costs in attentional and/or motor control. Moreover, from an applied perspective the present work suggests that passive exercise performed with a sedentary occupation may serve to support increased task-based vigilance and high-level EF performance.

### 4.5 Steady state changes in middle cerebral artery velocity do not predict simultaneous or postexercise executive function benefits

Some work has reported that active and passive exercise-mediated changes in MCAv are associated with the magnitude of postexercise EF benefits (Lucas et al., 2012; Kleinloog et al., 2019; Tari et al., 2020; Shirzad et al., 2022). Such results have been taken to evince that an increase in CBF provides the thermo-mechanical changes necessary to support improved EF. In the present study, we found that baseline to steady-state changes in CBF were not associated with simultaneous or post-intervention EF performance in either exercise conditions. It is, however, important to recognize that other work has shown that an improvement in EF is observed in prolonged exercise protocols that *decrease* CBF (Ogoh et al., 2014). Moreover, Washio and Ogoh (2023) have argued that individual differences in an exercise-mediated pressor response plays a more salient role in simultaneous and post-intervention EF benefits than CBF. Hence, it is likely that an exercise-mediated EF benefit does not underlie a unitary neurophysiological change (e.g., CBF); rather, it is likely accrued from interdependent processes that include CBF and pressor response changes as well as increased biomolecule availability (e.g., nitric oxide, brain-derived neurotrophic factor, catecholamines) (Zouhal et al., 2008; Knaepen et al., 2010; Pertiwi et al., 2015) and improved functional connectivity in EF networks (Schmitt et al., 2019).

### 4.6 Study limitations

We recognize our study is limited by at least four methodological traits. First, only healthy and physically active young adults participated in this study. As a result, it is uncertain whether our conclusions can be extended to older adults with reduced regular physical activity who demonstrate a cardiorespiratory and neurovascular reactivity to exercise distinct from healthy young adults (see also Chang et al., 2012; McLeod and Stromhaug, 2017). Second, the antipointing task employed here primarily evaluated the inhibitory control component of EF, and as such, future work should establish whether simultaneous passive exercise benefits each core component of EF (i.e., inhibitory control, working memory and cognitive flexibility. Third, we used only a single passive and active exercise duration (i.e., 20-min), and the active exercise condition was completed at a light intensity. Thus, it is unclear whether longer—or shorter—durations may differentially influence simultaneous and post-intervention benefits across passive and active exercise conditions, and we are unable to assert whether increased intensity during active exercise may have contributed the condition's null simultaneous EF benefit. Indeed, addressing whether the duration (i.e., 10-, 20-, 30-min) of passive exercise influences simultaneous and postexercise EF benefits is an important area and may provide foundational knowledge to support cognition and brain health in persons with reduced or absent mobility (e.g., individuals with hemiparesis or spinal cord injury). Moreover, future work should examine whether passive exercise duration and intensity influences not only simultaneous EF benefit but also the temporal persistence of postexercise EF benefits. Third, future work should include an explicit attentional probe during a simultaneous assessment to determine the extent by which dual-task interference influences EF task performance during passive and active exercise. Last, the TCD measure used here did not evaluate vessel diameter. This is a potential limitation given MCA dilation and constriction that has been observed under hypercapnic environments (Coverdale et al., 2015). That said, the literature provides consensus agreement that MCAv provides a valid estimate of an exercise-mediated increase in CBF (for review see, Smith et al., 2021).

### 4.7 Conclusions

Twenty minute single bouts of passive and active cycle ergometry provide an immediate postexercise EF benefit independent of exercise-mediated changes in CBF. Notably, however, only passive exercise supported a simultaneous EF benefit and is a finding we attribute to the condition's reduced task-based attentional demands compared to active cycle ergometry.

## Data availability statement

The raw data supporting the conclusions of this article will be made available by the authors, without undue reservation.

## Ethics statement

The studies involving humans were approved by Health Sciences Research Ethics Board, Western University. The studies were conducted in accordance with the local legislation and institutional requirements. The participants provided their written informed consent to participate in this study.

## Author contributions

CD: Writing – review & editing, Investigation, Formal analysis, Conceptualization. CE: Writing – review & editing, Investigation, Formal analysis. BT: Writing – review & editing, Methodology, Investigation, Formal analysis, Conceptualization. MH: Writing – review & editing, Writing – original draft, Supervision, Software, Methodology, Funding acquisition, Conceptualization.
